# Complication imaging after laparoscopic Roux-en-Y gastric bypass: clues to the diagnosis and pitfalls

**DOI:** 10.1186/s13244-026-02231-6

**Published:** 2026-03-04

**Authors:** Camilla Gebauer, Helmut Kopf, Christiane Kulinna-Cosentini, Georg Tentschert, Raphael Schima, Alexander Klaus, Wolfgang Schima

**Affiliations:** 1 Department of Diagnostic and Interventional Radiology, Barmherzige Schwestern Krankenhaus, Goettlicher Heiland Krankenhaus and Sankt Josef Krankenhaus, Vienna, Austria; 2 Sigmund Freud Private University, Vienna, Austria; 3 Department of Surgery, Barmherzige Schwestern Krankenhaus, Vienna, Austria; 4 Medical University of Vienna, Vienna, Austria

**Keywords:** Postoperative complications, Laparoscopic Roux-en-Y gastric bypass, Multidetector computed tomography, Anastomotic leak, Internal hernia

## Abstract

**Abstract:**

Obesity is a complex chronic disease with a rising global prevalence and significant health implications. The laparoscopic Roux-en-Y gastric bypass (LRYGB) is one of the most widely performed bariatric procedures worldwide, ensuring significant weight loss and reducing obesity-related comorbidities. However, the risk of postoperative complications remains considerable. Multidetector computed tomography (MDCT) is regarded as the primary imaging modality for the assessment of suspected complications, due to its high diagnostic accuracy. This review offers a comprehensive overview of early (≤ 30 days) and late (> 30 days) postoperative complications, including anastomotic leak, abscess, hemorrhage, small bowel obstruction (SBO), internal hernia, gastro-gastric fistula, intussusception, and marginal ulcer, with emphasis on characteristic MDCT features. Due to its advantage as a dynamic method, upper gastrointestinal (Gl) studies with oral contrast material may be helpful for the diagnosis of leak and gastro-gastric fistula formation. A comprehensive understanding of the altered postoperative anatomy and the specific radiological signs of complications are essential for accurate MDCT interpretation, minimizing diagnostic errors and enabling timely, targeted clinical intervention. Today, MRI can be considered a problem-solver through its possibility of combining static with dynamic sequences in selected cases. In this narrative review, we highlight the most frequent complications of Roux-en-Y gastric bypass (LRYGB), allowing radiologists to become familiar with the typical radiological features and pitfalls in MDCT, upper GI studies, and MRI, when facing this type of surgery.

**Critical relevance statement:**

Postoperative complications following laparoscopic LRYGB can pose considerable diagnostic challenges. Although MDCT is the most important modality, upper GI studies (for leakage or suspected gastro-gastric fistula) and increasingly MRI (for pouch problems or in pregnant patients) can improve diagnostic accuracy and support effective clinical decision-making.

**Key Points:**

- LRYGB complications are challenging due to altered anatomy and distinct imaging features.
- Postoperative bleeding, leaks with/without abscess, small bowel obstruction, and internal hernia are the most common serious complications.
- MDCT evaluation and reporting should be structured and focus on characteristic CT signs to support accurate imaging diagnosis.

**Graphical Abstract:**

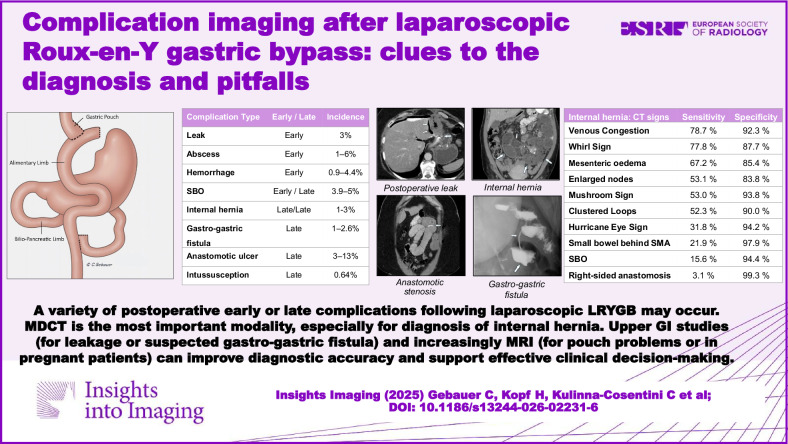

## Introduction

Severe obesity is a multifactorial and complex disease associated with a wide variety of comorbidities (such as type 2 diabetes, hypertension, and sleep-disordered breathing), which result in an overall diminished quality of life and substantially increase morbidity and mortality.

Since 1990, the prevalence among adults has more than doubled to 16% according to recent data from the World Health Organization (WHO) [1, 2]. According to the updated guidelines of the International Federation for the Surgery of Obesity and Metabolic Disorders (IFSO) and the American Society of Metabolic and Bariatric Surgery (ASMBS), bariatric surgery is recommended for patients with grade II obesity (body mass index (BMI) 35–39.9 kg/m²), regardless of the presence of comorbidities. It may also be considered in individuals with grade I obesity (BMI 30–34.9 kg/m²), who have not achieved substantial weight loss with non-surgical treatment. In addition, the guidelines advise lower BMI thresholds for Asian populations, defining clinical obesity as a BMI > 25 kg/m² and recommending consideration of bariatric surgery at a BMI > 27.5 kg/m² [3, 4]. Sleeve gastrectomy (SG) continues to be the most prevalent bariatric surgery globally, followed by the laparoscopic Roux-en-Y gastric bypass (LRYGB) [3]. LRYGB is regarded as one of the most effective treatments for morbid obesity, offering substantial and sustained weight loss along with improvement or resolution of related comorbidities [5]. Nevertheless, postoperative complications can arise, causing significant morbidity and even postoperative mortality [6]. A recent randomized comparison between SG and LRYGB did not show a statistically significant difference in complications rates between SG and LRYGB, but the spectrum of complications is quite different [7]. However, neither the guidelines of IFSO/ASMBS nor of the European Association for Endoscopic Surgery (EAES) on bariatric surgery address postoperative imaging of complications [4, 8]. Given the wide spectrum and the potential severity of postoperative complications, several imaging modalities, including multidetector-CT, fluoroscopy, and increasingly magnetic resonance imaging (MRI), play a central role.

Understanding the strengths and limitations of different imaging techniques is fundamental for optimizing patient outcomes after LRYGB. The aim of this review is to provide the typical imaging spectrum of complications, clues to subtle imaging findings, and pitfalls to be avoided.

## Surgical technique in LRYGB

**Fig. 1:**
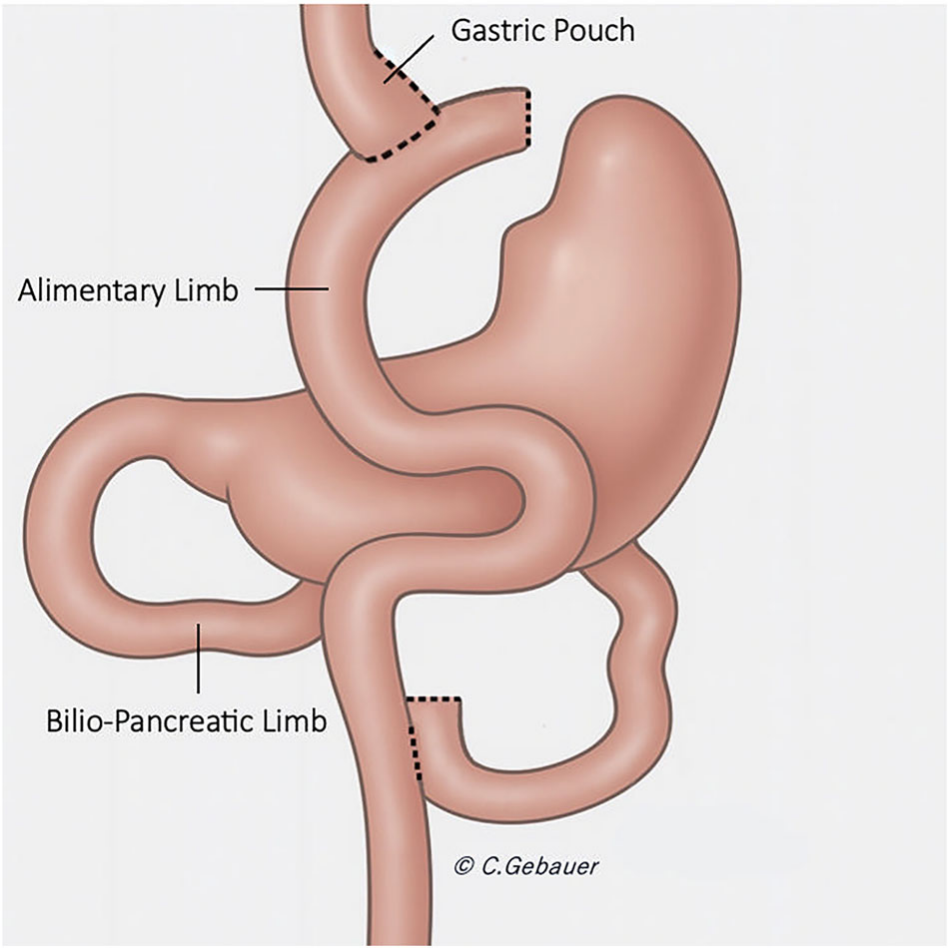
Illustration of Roux-en-Y gastric bypass procedure. The small gastric pouch is anastomosed with a jejunal loop (alimentary limb). The excluded stomach and the bilio-pancreatic limb are excluded from food passage. The alimentary limb and the bilio-pancreatic limb are anastomosed to form the common limb

The LRYGB involves the creation of a small stomach pouch and two small bowel limbs. The proximal portion of the stomach is shaped into a pouch, which typically has a volume of 25–40 mL [9, 10]. Two limbs of the small bowel are then constructed: the biliopancreatic limb, which is approximately 100 cm long and carries bile and pancreatic secretions from the excluded duodenum; and the alimentary (Roux) limb, which is typically 100–150 cm long and carries ingested food from the gastric pouch (Fig. 1). These limbs are then joined by a side-to-side jejunojejunostomy, which allows for the combination of digestive enzymes with ingested food [9, 10]. The gastric pouch is anastomosed in an end-to-side manner to the alimentary (Roux) limb of the jejunum [9, 10]. This “candy cane” configuration facilitates the direct passage of ingested food from the pouch to the small intestine while bypassing the gastric remnant, duodenum, and a portion of the proximal jejunum [9, 10].

## Different imaging modalities for diagnostic work-up

In many institutions, routine postoperative upper GI fluoroscopy with iodine-based contrast medium has been abandoned, but it may be crucial in suspected early complications. This technique is particularly useful for detecting early postoperative complications, such as leak, dehiscence, or anastomotic stricture, which may become more difficult to recognize in later phases of the examination due to overlying, contrast-filled small bowel loops [9, 11].

Contrast-enhanced multidetector computed tomography (MDCT) scan of the abdomen is the main pillar in the imaging of postoperative complications. Because of radiation considerations, only a single portal-venous phase scan is sought in our institution, except under special circumstances (i.e., suspected intraluminal bleeding). The use of positive vs. negative oral contrast has been a matter of debate in the radiology literature [12]. We include in our obesity surgery CT protocol overall 500 mL of dilute iodine-based contrast (1:50) administered orally approximately 20 min prior to scanning. Other groups would wait 30–60 min after oral contrast to start scanning [13].

MRI has recently gained more attention for imaging of postoperative complications. Dynamic swallowing MRI allows assessment of pouch size and position [14]. MRI using unenhanced pulse sequences (T2-w, T1-w, and diffusion-weighted imaging [DWI]) depicts the course of small bowel limbs. It has been shown to have an excellent sensitivity to detect internal hernia in pregnant patients [15].

Ultrasound is of limited value in the early postoperative setting to detect or rule out abdominal complications, given the high BMI of patients.

## Normal postoperative LRYGB imaging findings

**Fig. 2:**
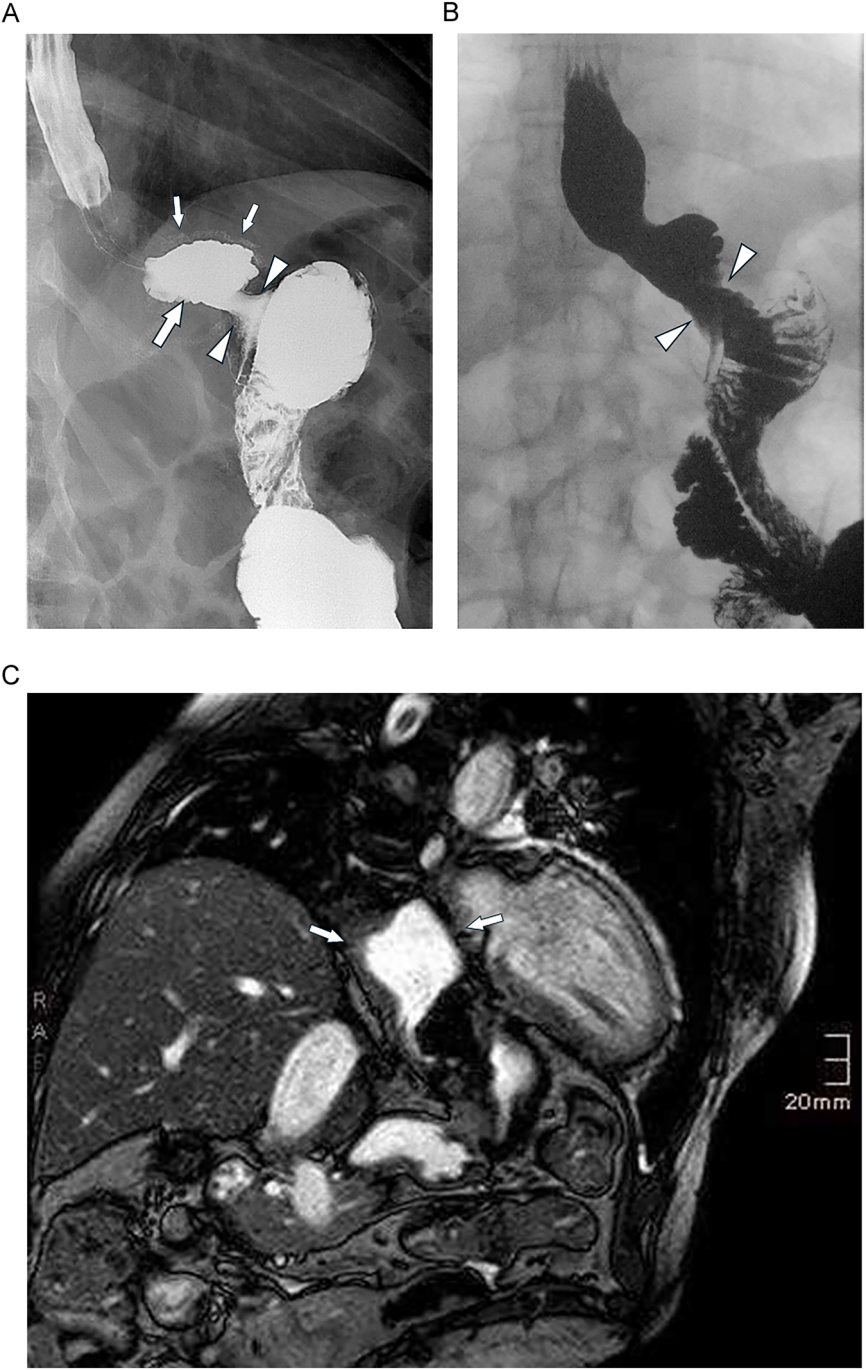
Normal postoperative anatomy at an upper GI study. **A** The gastric pouch is demonstrated (large arrow) with surgical staples along the contour (small arrows). The width of the gastro-jejunostomy anastomosis (arrowheads) cannot be assessed on this static image. **B** The cine sequence during swallowing shows normal widening of the anastomosis (arrowheads). **C** Dynamic MRI using a steady-state free precession sequence (True-FISP) shows the size and position of the pouch (arrows) after administration of oral contrast

The gastric pouch on an upper GI study appears typically as a small, round structure with a volume of app. 30 mL (with considerable variation according to surgical technique). The gastro-jejunal anastomosis should be visualized in two planes (anteroposterior and in profile). This allows visualization of gastric pouch size and the gastro-jejunal anastomosis diameter, often facilitated by the presence of surgical staples that delineate its anatomical borders (Fig. 2) [16]. It is important to follow the head of the contrast agent column as it enters the pouch and then passes the anastomosis, because real-time viewing of contrast extravasation facilitates diagnosis of staple line breakdown [17]. Dynamic MRI may also be used to assess the size and position of the pouch (Fig. 2).

**Fig. 3:**
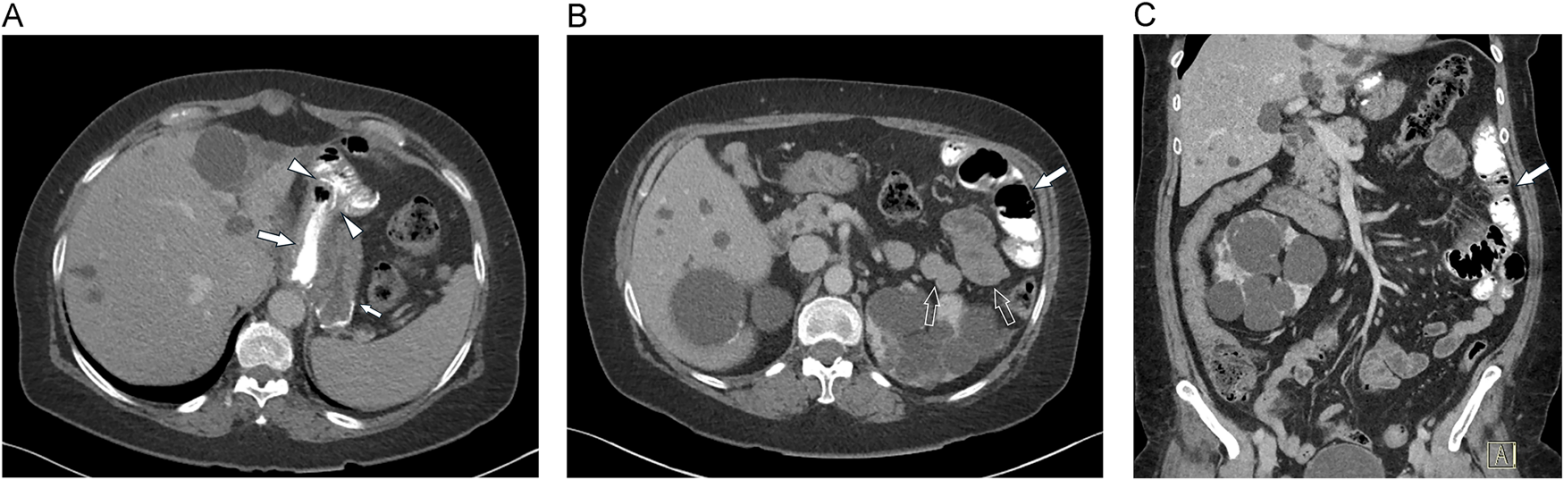
Normal postoperative anatomy at MDCT. Axial images show (**A**) the contrast-filled gastric pouch (arrow), the gastro-jejunostomy (arrowheads), the non-contrast-filled excluded stomach (small arrow), and (**B**) the contrast-filled alimentary limb (arrow), as well as the non-contrasted bilio-pancreatic limb (hollow arrows). **C** The coronal MPR shows the alimentary limb filled with orally administered contrast agent (arrow) and the normal course of the SMV and its tributaries. Assessment of the SMV on coronal MPR or MIP is critical for the diagnosis of an internal hernia

At CT, the administration of positive oral contrast enables excellent delineation of key post-surgical components, including the gastric pouch, gastrojejunostomy, and contrast-filled alimentary limb (in contrast to the non-contrast-filled bilio-pancreatic limb) (Fig. 3) [9]. The excluded residual stomach typically appears collapsed and must not be opacified (contain positive oral contrast). The jejuno-jejunostomy (highlighted by surgical staples seen at CT) is located in the midline to the left mid-abdomen. Identification of the jejuno-jejunostomy at CT can be important for the diagnosis of the obstruction site in SBO and in internal hernia (where the staples of the anastomosis, together with small bowel loops, may be found in an abnormal position). It is also key to visualize the superior mesenteric vein (SMV) and its tributaries to rule out an internal hernia with its typically abnormal course of mesenteric vessels (Fig. 3).

## Postoperative complications

Postoperative complications are categorized into two distinct categories: early (within the initial 30 days following surgery) and late (manifestations beyond 30 days) [18, 19]. Early complications commonly manifest in the first 7 days, while late complications may emerge even after years [20]. Early postoperative complications include bleeding, anastomotic insufficiency, leakage, intra-abdominal abscess, ischemia, and small bowel obstruction (SBO) due to trocar hernia or anastomotic stenosis. Early complications lead more often to surgical revision than late complications. Types of complications, temporal occurrence, and incidence according to the literature are shown in Table 1.

**Table 1:** Classification, onset, and incidence of postoperative complications [9, 16, 18, 21–27, 32, 37, 44, 45, 49]

Complication type	Early/late	Incidence
Leak	Early	3%
Abscess	Early	1–6%
Hemorrhage	Early	0.9–4.4%
SBO	Early/late	3.9–5%
Internal hernia	Late/late	1-3%
Gastro-gastric fistula	Late	1–2.6%
Anastomotic ulcer	Late	3–13%
Intussusception	Late	0.64%

## Early postoperative complications

### Hemorrhage

**Fig. 4:**
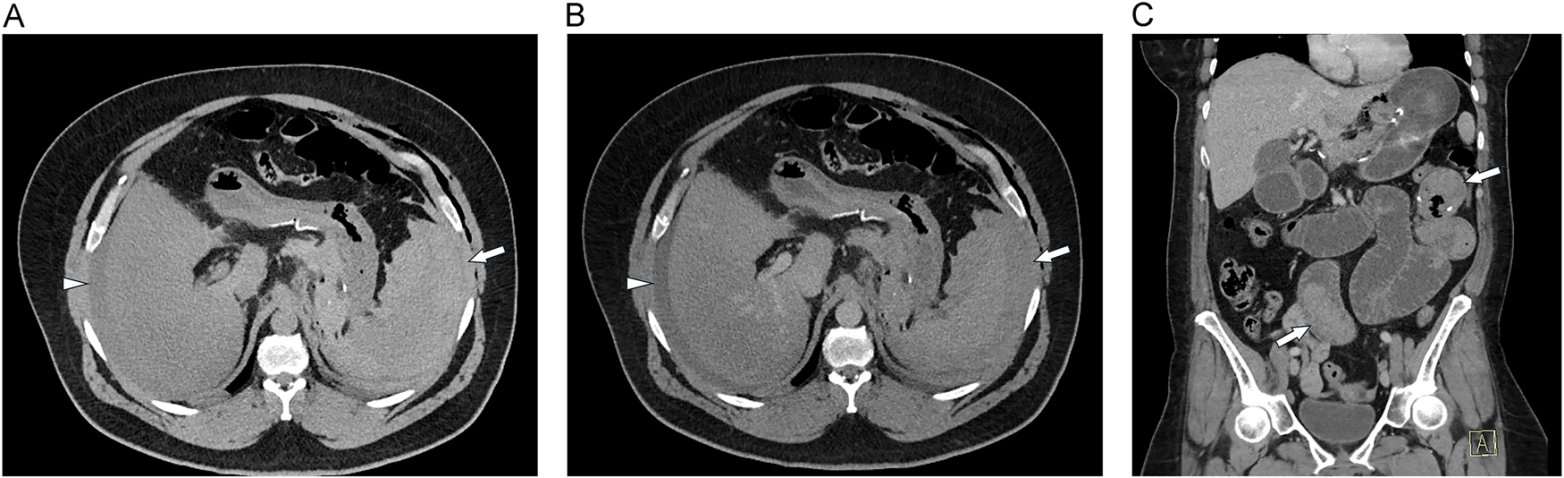
Hemorrhage. **A** Unenhanced and (**B**) portal-venous phase MDCT images show extraluminal hyperdense hematoma mainly around the spleen (large arrows) and the gastric pouch (small arrow). There is also hemorrhagic ascites (arrowheads) around the right lobe of the liver. Oral contrast was avoided in order not to mask possible contrast extravasation due to bleeding. **C** Intraluminal hemorrhage in another patient: coronal MPR of MDCT shows large hyperdense clots in the biliopancreatic limb (arrows)

Postoperative hemorrhage is a significant early complication, typically occurring within the first 48 h [21]. Current literature reports a range of incidence rates from 0.9% to 4.4%, with more recent studies indicating a downward trend [21–26]. The etiology of hemorrhage is multifactorial, with intraluminal bleeding potentially resulting from staple line bleeding or the presence of an acute marginal ulcer at the gastrojejunostomy. Conversely, extraluminal bleeding may originate from intra-abdominal sources or port sites. Of 60–72% of hemorrhages are intraluminal and 28–40% extraluminal (Fig. 4) [23]. In the acute setting, a hematoma on CT shows high attenuation, and IV contrast material extravasation may be seen in severe cases. However, multi-phasic contrast-enhanced CT protocols to facilitate bleeding detection have to be carefully considered because of radiation concerns [12]. The vast majority of cases (68–75%) could be managed conservatively using interventions such as blood transfusions, drainage, coagulation management, and pharmacological therapy; only 15% require revision surgery [25]. Late postoperative bleeding is rare (app. 1%) and has been shown to be always intraluminal [24]. In surgical series, diagnostic evaluation includes esophagogastroscopy and, selectively, CT, especially if extraluminal bleeding is suspected [24].

### Anastomotic insufficiency and leak

Postoperative anastomotic insufficiency or leak represents a significant complication following LRYGB, with a reported incidence of up to 30% of procedure-related mortality [27]. Leaks occur in approximately 3% of patients within the first 10 days post-surgery [9, 28]. By far the most common site of leakage is at the gastro-jejunostomy, although other potential sites include the proximal jejunal stump, distal portion of the gastric pouch, and jejuno-jejunostomy [11]. Clinically, affected patients typically present with abdominal pain, fever, and tachycardia, making an urgent diagnosis key to preveni prevent severe sequelae such as abscess formation or peritonitis [9]. The upper GI study is a fast and inexpensive test, but it has been shown to be inferior to CT with a detection of only 30% (vs 56% for CT) [29]. A pitfall is the upper GI study performed too early: In a small series, upper GI studies on postoperative Day 1 detected none of the anastomotic leaks, which presented then clinically on postoperative day 2–4 [30].

**Fig. 5:**
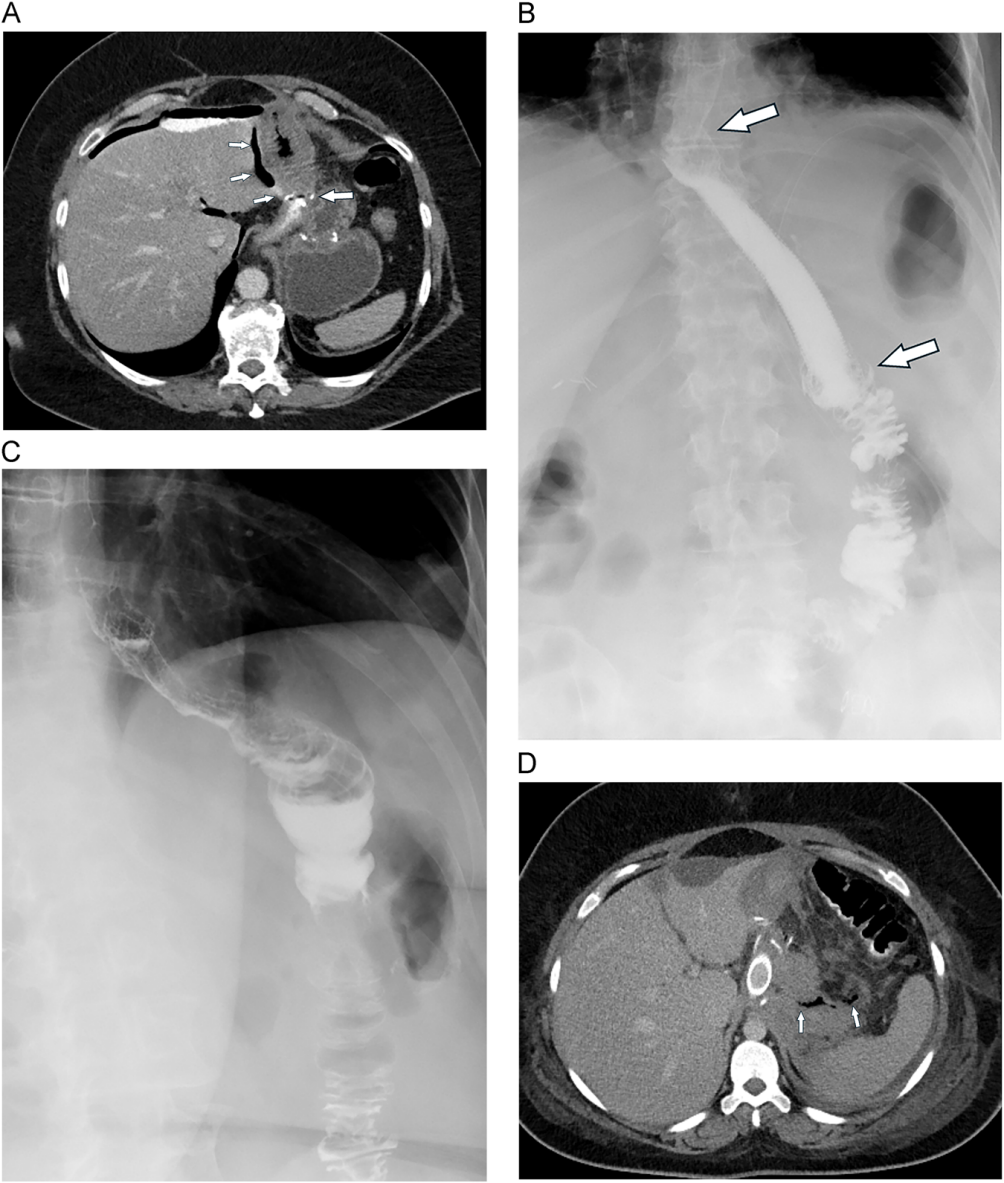
Anastomotic leak in a patient with postoperative fever and increasing CRP. **A** MDCT shows extravasation of orally administered contrast and air (small arrows) at the anastomosis (large arrow). There is a lot of free air and contrast anterior to the liver. **B** Upper GI study after surgical revision and endoscopic stent placement to seal the leak shows good stent positioning and no extravasation. Arrows indicatethe upper and lower ends of the stent. **C** Follow-up study after stent removal shows unremarkable findings. **D** Pitfall: MDCT 2 days after revision surgery and stent placement still shows some extraluminal gas bubbles (arrows), which might be seen as a persistent leak. However, a leak could be ruled out during follow-up

The preferred imaging modality is contrast-enhanced CT, which can reveal typical and specific findings such as extravasation of oral contrast (Fig. 5). MDCT with oral and IV contrast has been shown to have a sensitivity of 89–100% and a specificity of 69-78%, with an excellent negative predictive value of 97–100% to rule out leakage. The presence of free intraperitoneal air can give a hint, especially if massive (Fig. 5). However, pitfalls are not uncommon: research has indicated that performing CT scans too early after surgery can significantly reduce the sensitivity of detecting leaks. This is particularly the case due to postoperative tissue swelling and the presence of microleaks that have not yet fully developed [31]. Vice versa, interpretation of extraluminal air in the first days after surgery may be difficult. Clinically non-significant extraluminal air is more likely isolated (not associated with free intraperitoneal air) and not clustered near an anastomosis, although, to the best of our knowledge, no CT study has systematically evaluated this issue (Fig. 5). Most postoperative leaks require antibiotic therapy in combination with drainage and/or stent placement (Fig. 5) [9].

### Abscess

**Fig. 6:**
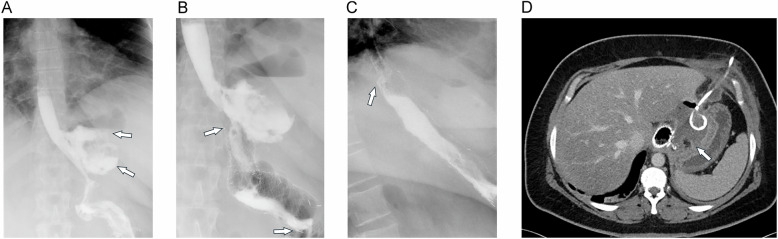
Anastomotic leak with abscess and CT-guided drainage. **A** Upper GI study shows large contrast extravasation at the anastomosis and a large extraluminal gas collection (arrows). **B** A stent was placed, which did not seal the leak due to distal migration (arrows indicate upper and lower ends of the stent). **C** After placement of a second (more proximal) stent (arrow indicates proximal end of new stent), no extravasation is seen. There are still some small residual distant extraluminal gas bubbles. **D** Due to persistent fever, MDCT was performed, which shows a large abscess (arrow), which was subsequently drained under CT-guidance

Postoperatively, abscesses typically develop around 10 days after LRYGB surgery and have an incidence of 1–6% [27]. Most commonly, they are secondary gastrointestinal (GI) tract leaks. Less frequently, they arise from contamination of undrained intra-abdominal fluid collections [27]. The management of these abscesses necessitates effective drainage of the infected fluid, which can be achieved surgically or percutaneously under CT guidance, if technically feasible. The efficacy of the therapeutic intervention is contingent upon the timely and accurate identification of the underlying leak (Fig. 6). However, too early CT may show leakage, but fail to show abscess formation.

### SBO

**Fig. 7:**
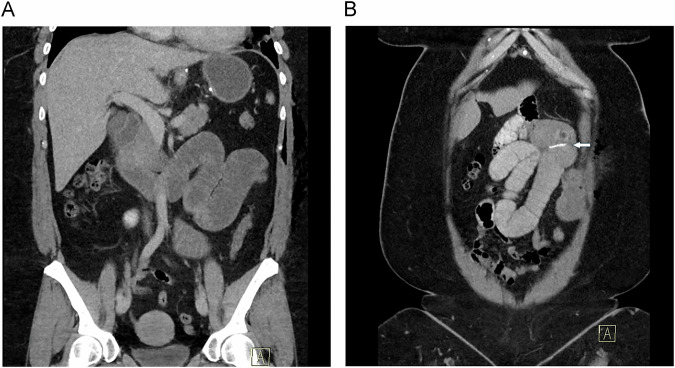
SBO due to early postoperative swelling of the jejuno-jejunostomy. Coronal MPRs of MDCT show typically (**A**) the dilated bilio-pancreatic limb and (**B**) the dilated alimentary limb (filled with oral contrast) leading to the jejuno-jejunostomy (indicated by surgical clips, small arrow). Distal bowel loops are collapsed

**Fig. 8:**
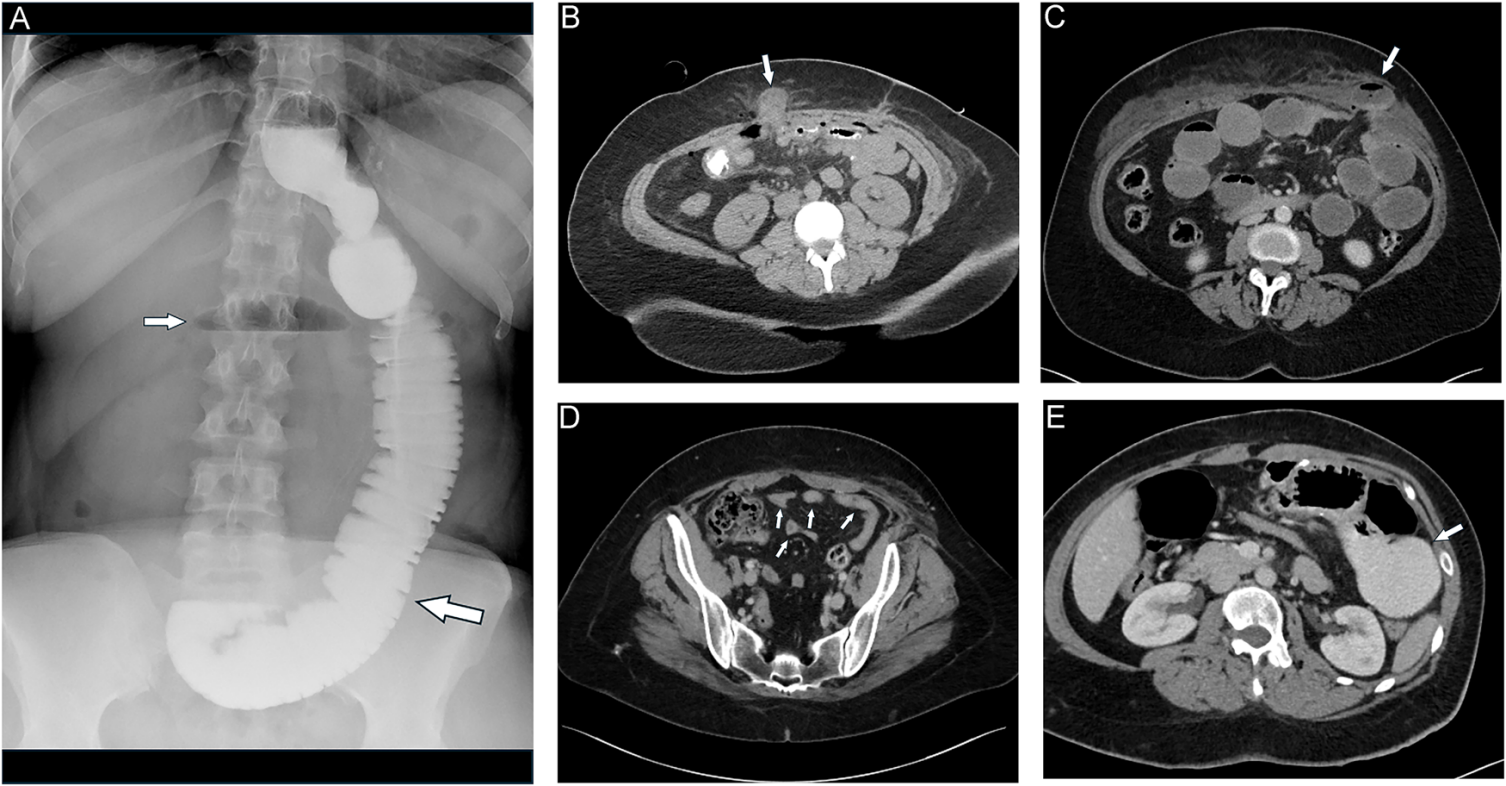
SBO due to trocar hernia. The patient developed pain one day after surgery. **A** The upper GI study shows a massively dilated jejunal loop (large arrow) with layering of contrast and a not yet contrast-filled small bowel loop with air-fluid level (small arrow). **B** MDCT performed immediately after shows a small bowel loop (large arrow) protruding through the abdominal wall at the site of a surgically placed trocar (arrow). **C**, **D** In another patient with postoperative ileus diagnosis is made straight with MDCT, (**C**) showing dilated small bowel loops leading to the loop herniated through the abdominal wall (large arrow) and (**D**) collapsed loops distally (small arrows). Oral contrast could not be given due to vomiting. **E** Pitfall: MDCT shows a massively dilated loop filled with contrast (arrow). However, there are no other dilated small bowel loops to indicate SBO. Diagnosis was a dilated blind loop adjacent to the side-to-side jejunostomy

SBO following LRYGB can occur as either an early or late complication [32]. The reported incidence of SBO ranges from 3.9% to 5% [9, 32]. Currently, SBO is categorized into three types: Type A involves the alimentary limb proximal to the jejuno-jejunostomy (e.g., due to trocar hernia of the abdominal wall) [33]. It is often diagnosed by fluoroscopic upper GI or CT studies, which demonstrate a dilated alimentary limb along with a collapsed bilio-pancreatic limb [9]. Type B affects the bilio-pancreatic limb proximal to the jejuno-jejunostomy. This is diagnosed by CT revealing a fluid-filled, distended excluded stomach and a dilated bilio-pancreatic limb, while the alimentary limb is of normal caliber [9, 34]. Type C refers to obstruction of the jejuno-jejunostomy (Fig. 8) or the common channel distal to it (e.g., due to adhesions), with imaging findings including dilatation of both alimentary and bilio-pancreatic limbs [9]. Early postoperative SBO is typically caused by swelling of the jejuno-jejunostomy (Fig. 7), incisional (trocar) hernia (Fig. 8), or surgical malalignment with kinking of the anastomosis [35]. In clinical practice, late occurrence of SBO is most likely caused by internal hernia (which may affect the alimentary and bilio-pancreatic limb alike) or adhesions. Mesocolic window stenosis may occur in retrocolic placement of the alimentary limb (through a surgically created defect in the transverse mesocolon) and can be seen in the early or late postoperative setting [33]. A retrospective study by Elms et al identified adhesions as the cause of SBO in 47.6% of reoperations, while internal hernias were found in 27.6% of cases [32].

### Ischemia

Ischemia in the alimentary limb of the jejunum represents another potential complication, which may occur either early or late in the postoperative course. The underlying pathophysiology is attributed to suboptimal surgical technique with tension in the mobilized distal jejunal segment, resulting in perfusion compromise and subsequent ischemia [9]. Clinical presentation varies and may include abdominal pain and upper GI bleeding. While mild jejunal ischemia can be self-limiting, severe cases can progress to bowel infarction [9]. Although acute ischemia is rare nowadays due to the standardization of surgical technique, imaging plays a key role in diagnosis. Fluoroscopy may reveal thickened mucosal folds due to edema or hemorrhage, while CT imaging demonstrates a thickened jejunal wall, edematous mesentery, and thrombosed mesenteric vessels—findings that may mimic enteritis [9]. Chronic ischemia can lead to the development of large ischemic ulcers within the alimentary limb. The postoperative detection of jejunal ulcers larger than 2.5 cm should raise suspicion for chronic ischemia and necessitate prompt initiation of medical therapy, such as proton pump inhibitors. In cases refractory to conservative treatment, surgical intervention must be considered [9].

## Late postoperative complications

### Internal hernia

Due to significant postoperative weight loss, mostly during the first year, the loss of the protective fat around the intestine, in combination with the laparoscopic approach (which may result in fewer adhesions around the bowel), can result in widening of postoperative defects in the mesentery.

**Fig. 9:**
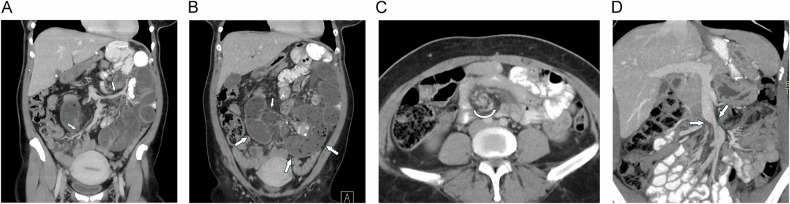
CT signs of internal hernia. **A** Coronal MRP shows deviated SMV with dilated tributaries (small arrows). **B** The small bowel loops in the mid-abdomen are dilated and C-shaped (large arrows), and there is mesenteric edema (small arrow). **C** In another patient, axial MDCT shows typical rotation of the mesenteric vessels around their axis (curved arrow) (“whirl sign”). **D** In the same patient, coronal MIP shows beak-like constriction of SMV due to torsion (arrows)

**Fig. 10:**
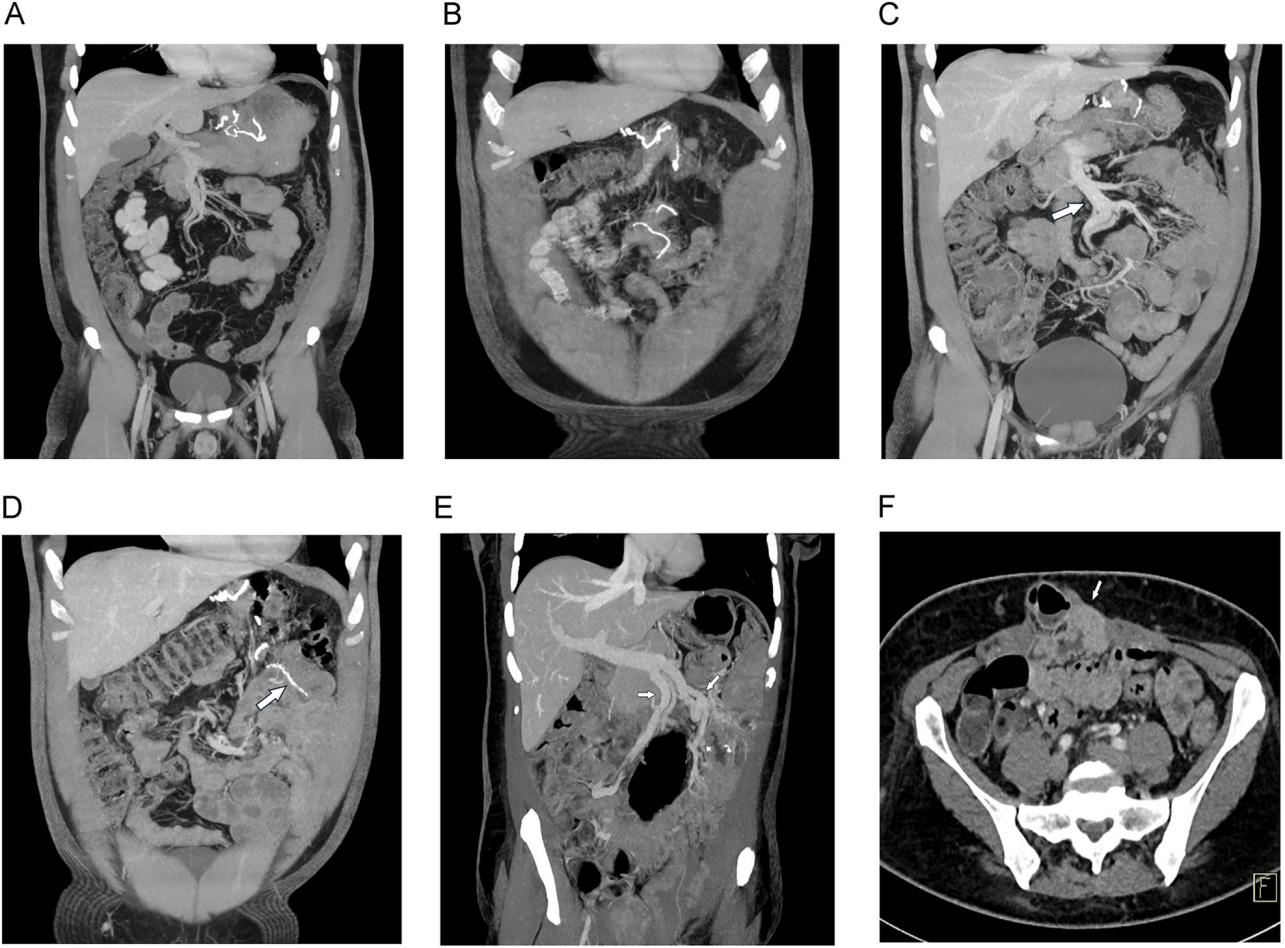
Internal hernia diagnosis during follow-up. **A** Coronal MIP shows normal course of SMV and (**B**) normal position of jejuno-jejunostomy (small arrow). Clinical course was uneventful. **C**, **D** In a follow-up examination a few months later (due to recurrent abdominal pain), there is (**C**) new displacement of the SMV and (**D**) of the anastomosis. Note that neither of the 2 CT studies shows small bowel dilatation. **E** Pitfall in a patient with a history of small bowel resection and extensive adhesions: There is severe displacement of the SMV (arrows), but no other signs of internal hernia are present. **F** MDCT also shows an incisional hernia (arrow), which was repaired with a mesh. No internal hernia was found

Following LRYGB surgery defects at three typical anatomical sites become the source of internal hernias: the transverse mesocolon in patients with a retrocolic gastrojejunostomy, where the alimentary limb is routed through a surgically created defect in the mesocolon (transmesocolic hernia); the small bowel mesentery at the site of the jejunojejunostomy (jejuno-jejunostomy defect or Brolin´s space); and the space behind the alimentary limb, known as Petersen’s defect (Petersen hernia) [9, 36]. These anatomical vulnerabilities are clinically significant, as internal hernias represent a potentially serious postoperative complication, with reported incidence rates ranging from 1% to 3% depending on the surgical technique [37]. In series, where surgical closure of mesenteric defects had not been performed, a much higher incidence of internal hernia has been reported than in institutions, where meticulous closure of mesenteric defects is sought. CT diagnosis of internal hernia can be very challenging: depending on the size of the defect, several small bowel loops may intermittently herniate through this defect, without imaging signs of strangulation. A meta-analysis encompassing 20 studies and a total of 1637 patients reported a pooled sensitivity of 82.0%, a specificity of 84.8% for CT-based diagnosis of internal hernias [38]. Despite these diagnostic limitations, some characteristic CT signs can be observed (Fig. 9): The “whirl sign” (or “swirl”) reflects a twisting of the mesentery, where mesenteric vessels spiral around a central axis, similar to the CT signs seen in small bowel volvulus [36]. This sign has been reported to be moderately sensitive (66%), but highly specific [38]. Twisting of the SMV may also result in a beak-like narrowing of the vein adjacent to the superior mesenteric artery (“beaking phenomenon”). The “clustered loop sign” describes the abnormal aggregation of small bowel loops within a confined area of the abdominal cavity, often appearing compressed, closely packed, and C-shaped. The “hurricane eye sign” results from rotation or displacement of intestinal loops around a central mesenteric vessel, creating a circular or semi-circular pattern reminiscent of a hurricane’s eye [36, 39]. An abnormal position of the jejuno-jejunostomy (as demonstrated by the surgical staples) (Fig. 10) in conjunction with SBO may further suggest the presence of an internal hernia [9, 36]. This sign has a very low sensitivity, but its specificity has been found to approach 100% [40]. Studies indicate that the venous congestion, whirl sign, and mesenteric edema (sensitivity of 78.7%, 77.8%, and 67.2%, respectively) are the radiological signs with the highest sensitivity [38]. In a study by Dilauro et al, mesenteric whirl (sensitivity 86–89%, specificity 86–90%) and the beaking phenomenon (sensitivity 80-88%, specificity 94–95%) demonstrated the highest diagnostic accuracy for CT-based detection of internal hernia following gastric bypass surgery [41]. A decision tree combining mesenteric whirl and SMO achieved the highest sensitivity (96%), while the combination of beaking phenomenon and bowel obstruction yielded the highest specificity (92%) (41). That decision tree model yielded the highest diagnostic odds ratio of 154 for the combination of whirl sign and SBO (41). Adding to this combination other imaging features such as criss-cross sign, right-sided anastomosis, and/or presence of small bowel behind the SMA slightly lowered the odds ratio to 151–128 (41). Systematic reviewing of CT signs and structured reporting can improve detection of internal hernia: Ederveen et al compared the diagnostic value of free text reporting with structured reporting using the 10 CT signs (as shown in Table 2) to make the diagnosis [40]. With structured reporting, accuracy rose from 87.2% to 93.1%, with positive predictive value even climbing from 55.6% to 81.3% [40].

**Table 2:** CT signs of internal hernia: definition, sensitivity, specificity [38, 40]

Signs	Definition	Sens. (95% CI)	Spec. (95% CI)
Venous congestion (syn.: SMV beaking)	Tapering of mesenteric veins with subsequent engorgement	78.7 (70.9–84.9)	92.3 (81.9–96.9)
Whirl sign	Twisting of the mesenteric vessels	77.8 (67.8–85.3)	87.7 (75.4–94.3)
Mesenteric edema	Haziness of the mesenteric fat	67.2 (57.0–76.0)	85.4 (70.6–93.4)
Enlarged nodes	Presence of enlarged mes. nodes as a secondary sign of lymphatic obstruction from mes. torsion	53.1 (35.8–70.4)	83.8 (77.7–89.9)
Mushroom sign	Herniated mesenteric root with protrusion of bowel between the SMA and its branches	53.0 (40.5–65.1)	93.8 (88.3–96.8)
Clustered loops	Abnormal aggregation of small bowel loops within a confined area of the abdominal cavity	52.3 (13.9–88.1)	90.0 (74.5–96.5)
Hurricane eye sign	Tubular or round shape of the distal mesenteric fat, closely surrounded by bowel loops	31.8 (13.3–58.7)	94.2 (83.0–98.2)
Small bowel behind the SMA	The small bowel, other than the duodenum, passes behind the SMA	21.9 (7.6–36.2)	97.9 (95.5–100)
SBO	Dilated small bowel loops with air-fluid levels	15.6 (3.0–28.2)	94.4 (90.6–98.2)
Right-sided anastomosis	Right-sided location of the distal jejunal anastomosis	3.1 (0–9.2)	99.3 (97.9–100)

Although it is advised to defer pregnancy after LRYGB surgery, the late occurrence of internal hernia may coincide with or be precipitated by pregnancy [42]. In retrospective series, Krishna S et al and van Berkel B et al evaluated non-contrast MRI (using T2w TSE and T1w GRE, and DWI or steady-state free precession pulse sequences) for the diagnosis of internal hernia [15, 43]. Sensitivity for different readers ranged from 75% to 100%. Presence of Whirl Sign, beaking of SMV, mesenteric vessels engorgement, and mesenteric edema were both sensitive and inter-reader reliable [15, 43].

### Gastric ulcer

A late complication is the development of gastric ulcers, especially anastomotic ulcers, which occur in approximately 3–13% of patients [9, 18]. These ulcers are primarily caused by chronic exposure of the jejunal mucosa in the Roux limb to gastric acid, with additional contributions from Helicobacter pylori infection. Treatment is generally conservative and consists of proton pump inhibitor therapy [9]. Chronic ulcers may also lead to anastomotic stricture at the gastro-jejunostomy, which may lead to gastric pouch dilatation and vomiting, typically occurring a few minutes after oral intake [16]. Only large ulcers can be seen at contrast-enhanced MDCT as non-transmural defects.

### Gastro-gastric fistula

**Fig. 11:**
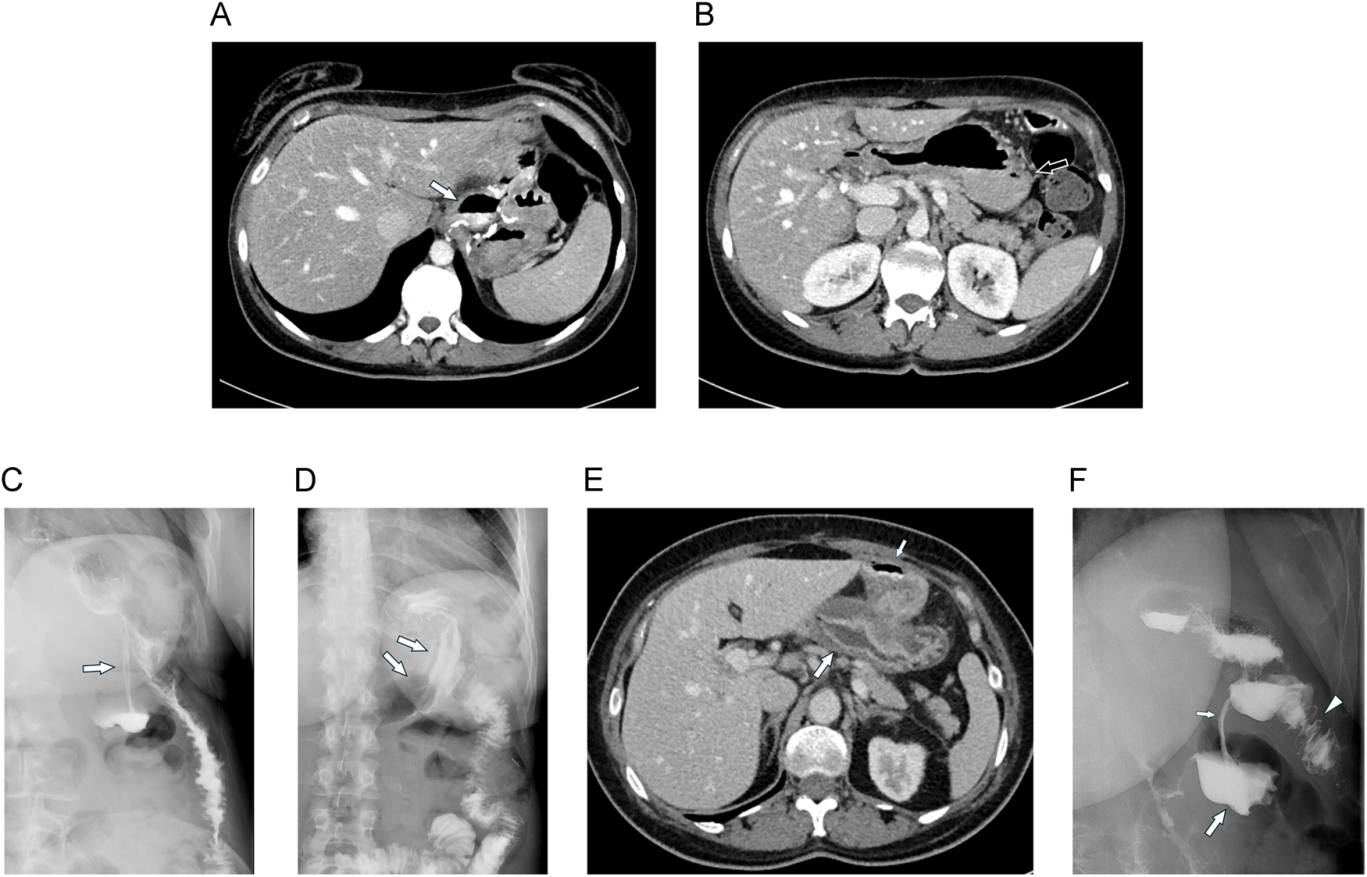
Gastro-gastric fistula. Contrast-enhanced MDCT with dilute oral contrast material shows (**A**) both the gastric pouch (large arrow) and (**B**) the excluded stomach (hollow arrow) filled with contrast, which is pathognomonic for gastro-gastric fistula. The upper GI study confirms the presence of a fistula. **C** In the upright position, contrast leakage from the anastomosis with fistula formation is seen (arrow). **D** In the supine position, the oral contrast spreads out in the excluded stomach, and typical gastric folds are delineated (arrows). **E** Pitfall: MDCT shows the excluded stomach with mucosal thickening and edema (large arrow). The alimentary limb is filled with oral contrast (small arrow), but not the excluded stomach. **F** Same-day upper GI study clearly shows the gastro-gastric fistula (small arrow) with filling of the excluded stomach (large arrow). Alimentary limb (arrowhead)

Gastro-gastric fistula is a rare complication, with an incidence of 1–2.6% [16, 44, 45]. However, the true incidence is probably underestimated, as asymptomatic patients may never undergo imaging [46]. It is defined as an abnormal connection between the gastric pouch and the excluded gastric remnant and may be caused by anastomotic insufficiency with fistula formation or ulceration of the gastric pouch with contained perforation into the excluded stomach [47]. This diagnosis should be considered in patients presenting with failure to sufficiently lose weight or even weight gain. However, in some patients, non-specific symptoms such as epigastric pain, vomiting, or hematemesis are found. Preferred diagnostic modalities include upper GI endoscopy, upper GI studies and CT [16, 44, 45]. The typical finding is the presence of contrast extravasation from the gastric pouch to the excluded stomach at fluoroscopy. At CT the appearance of positive oral contrast in the excluded stomach is pathognomonic, even if the fistula cannot be revealed on multi-planar reconstructions (Fig. 11). However, CT with oral contrast is not infallible and upper GI studies may occasionally show fistula not seen with CT (Fig. 11). Thus, both methods must be considered complementary in cases with high suspicion of gastro-gastric fistula and negative endoscopy. In general, CT with oral contrast has been demonstrated to exhibit a diagnostic accuracy comparable to that of upper GI studies and endoscopy in the detection of gastro-gastric fistulas after LRYGB, with a sensitivity of 73.1% and a specificity of 95.2% [48].

### Intussusception

**Fig. 12:**
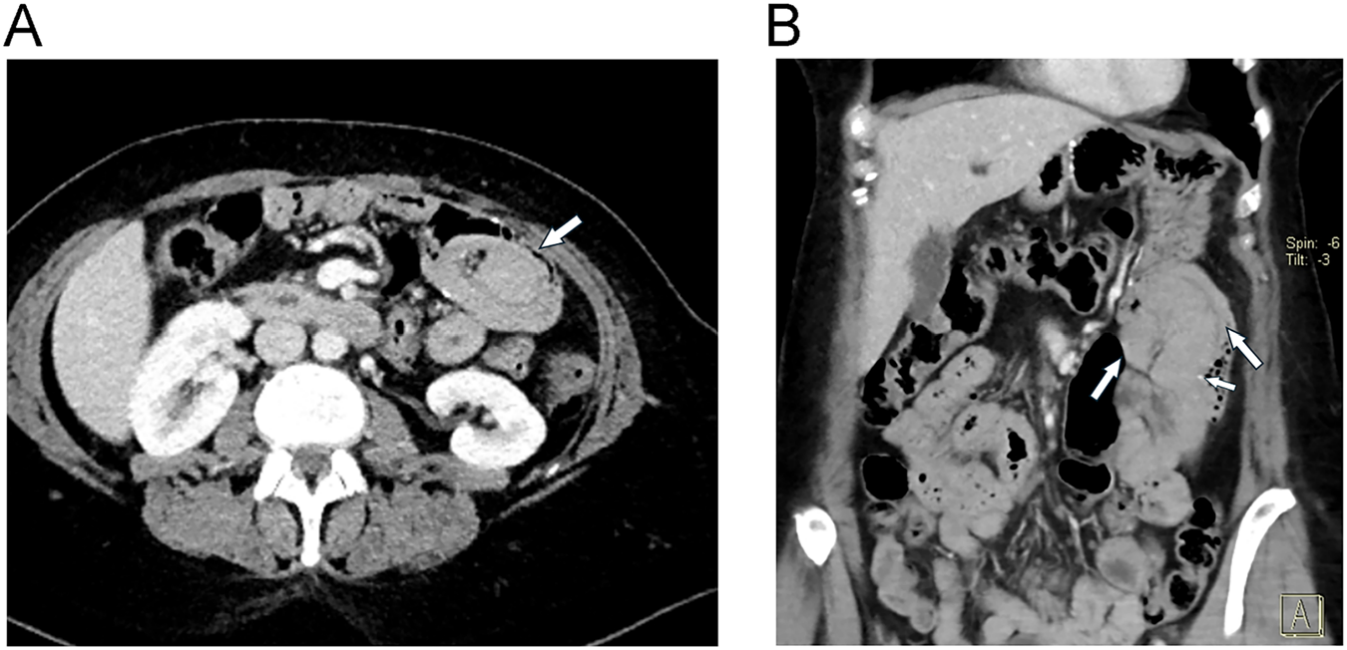
Transient intussusception. **A** The axial MDCT image shows the typical target sign appearance of the intussuscepted small bowel loop (arrow). **B** In the coronal MPR, the intussusception can be seen to be retrograde (arrows). Surgical staples (small arrow) indicate the level of the jejuno-jejunostomy (anastomosis)

Intussusceptions represent a very rare (incidence 0.64%) and late complication with a median onset of approximately 52 months postoperatively [49]. Although intussusception is an extremely uncommon occurrence, recurrence after an initial onset is not infrequent. Recent meta-analyses and systematic reviews report recurrence rates ranging from 7.7% to 22% [49–51]. Risk factors include female sex, accounting for 98% of cases, and rapid, significant postoperative weight loss [49, 51]. Unlike in the general population, where intussusception is typically anterograde (isoperistaltic, with the proximal loop telescoping into the distal loop), patients following LRGB are more likely to experience retrograde intussusception (Fig. 12) [5, 51]. Intussusception can lead to further complications, including SBO, bowel ischemia, infection, and necrosis [51]. A retrospective study found that in LRYGB patients, a CT-detected intussusception length > 100 mm strongly predicted SBO requiring surgery, with 80–100% sensitivity and 86–93% specificity. Furthermore, proximal bowel dilatation was found to correlate with obstruction, thus underscoring the utility of CT in identifying cases with clinical significance [52]. While the exact pathophysiology remains unclear, several theories have been proposed [51]. One suggests that the staple line at the anastomosis may serve as a lead point [51, 53]. Another hypothesis suggests that disruption of the duodenal pacemaker cells due to jejunal transection during surgery may result in ectopic pacemaker activity in the alimentary limb, triggering abnormal peristalsis and retrograde intussusception [51, 53]. Furthermore, significant weight loss may lead to elongation and thinning of the mesentery, reducing its mechanical resistance and increasing susceptibility to intussusception [51].

### Pouch migration

Intrathoracic pouch migration after LRYGB can be seen in 5–10%, where the gastric pouch moves into the chest [54]. Symptoms like regurgitation, reflux, chest pain, or weight regain can be the consequence. The gastric pouch should be examined in multiple planes, either in CT or MRI, to measure the exact volume. Intrathoracic pouch migration could be diagnosed, if staple lines are visible above the hiatus. A recent study demonstrated that MRI is a valuable non-ionizing alternative method to 3D-CT in the measurement of volume and pouch migration [14].

#### Other complications

After LRYGB, other uncommon complications may also occur: in candy cane syndrome, a redundant afferent Roux limb near the gastrojejunostomy may cause food stasis and mechanical irritation [55]. Diagnosis is made by upper GI series and endoscopy. Acute mesenteric venous thrombosis is a potentially catastrophic early complication [56]. Etiology is varied, with prothrombotic states due to congenital hypercoagulability, oral contraceptives, inflammatory diseases, or a history of recent abdominal surgery being most common. The incidence of post-bariatric surgery mesenteric venous thrombosis is 0.4%, with a higher incidence after SG than after LRYGB [57]. The diagnosis is made with contrast-enhanced CT, which reliably demonstrates the extent. Other complications after LRYGB, such as bile reflux or dumping syndrome, may affect quality of life considerably, but they are not in the domain of radiologic diagnosis.

## Conclusion

A wide spectrum of potential complications may occur early or later after LRYGB surgery. Even with profound knowledge of postsurgical anatomy, complications can pose a diagnostic challenge. MDCT with oral contrast is the main diagnostic pillar in suspected early complications (leak without/with abscess, bleeding, SBO, etc.). Several CT signs, such as the whirl sign, SMV beaking (venous engorgement), mesenteric edema, clustered loops, and SBO, have been shown to present good to excellent sensitivity for the detection of internal hernia [58]. Adoption of a structured reporting approach increases the diagnostic yield of CT. Upper GI Studies with iodine-based contrast are employed for the detection of anastomotic leak and gastro-gastric fistula. Recently, non-contrast MRI has gained recognition for the detection of LRYGB complications, especially internal hernia in pregnant patients.

## Data Availability

Imaging material from fluoroscopic, CT, and MRI studies is available on the PACS of the contributing institutions.

## Abbreviations

BMI: Body mass index
GI: Gastrointestinal
LRYGB: Laparoscopic Roux-en-Y gastric bypass
MDCT: Multidetector computed tomography
MRI: Magnetic resonance imaging
SBO: Small bowel obstruction
SG: Sleeve gastrectomy
SMV: Superior mesenteric vein
